# Molecular Biomarker Testing in Kidney Cancer: Integrating Diagnosis, Prediction, and Emerging Renal Tumor Entities

**DOI:** 10.3390/cancers18203341

**Published:** 2026-10-16

**Authors:** Busra Yaprak Bayrak, Elif Ozsagir, Nazif Alperen Yıldırım, Kerem Teke, Michael Cheng, Anna Caliò, Guido Martignoni, Mahmut Akgul, Liang Cheng

**Affiliations:** 1 Department of Pathology, School of Medicine, Kocaeli University, Kocaeli 41001, Turkey; busra.yaprakbayrak@kocaeli.edu.tr (B.Y.B.); elif.ozsagir@kocaeli.edu.tr (E.O.); 2 Department of Pathology, Gulhane Research and Training Hospital, Ankara 06010, Turkey; nazifalperen.yildirim@sbu.edu.tr; 3 Department of Urology, School of Medicine, Kocaeli University, Kocaeli 41001, Turkey; kerem.teke@kocaeli.edu.tr; 4 Department of Pathology, Duke University School of Medicine, Durham, NC 27710, USA; michael.cheng@duke.edu; 5 Section of Pathology, Department of Diagnostics and Public Health, University of Verona, 37134 Verona, Italy; anna.calio@univr.it (A.C.); guido.martignoni@univr.it (G.M.); 6 Department of Pathology, Pederzoli Hospital, 37019 Peschiera, Italy; 7 Department of Pathology, School of Medicine, Atlas University, Istanbul 34408, Turkey; makgul@bwh.harvard.edu; 8 Department of Pathology, Brigham and Women’s Hospital, Boston, MA 02115, USA; 9 Department of Pathology and Laboratory Medicine, Department of Surgery (Urology), Brown University Warren Alpert Medical School, the Legorreta Cancer Center at Brown University, and Brown University Health, Providence, RI 02912, USA

**Keywords:** kidney tumors, renal cell carcinoma, molecular biomarkers, renal pathology, immunohistochemistry, next-generation sequencing, hereditary renal tumors, predictive biomarkers, WHO classification

## Abstract

**Background**: Kidney tumors comprise a biologically diverse and diagnostically challenging group of neoplasms. Many entities share overlapping morphologic features despite having distinct molecular profiles. Although histomorphology remains the cornerstone of diagnosis, molecular biomarker testing has become increasingly important for accurate classification, recognition of emerging entities, identification of hereditary cancer syndromes, and therapeutic decision-making. **Focus of the Review**: This narrative review provides a practical, pathology-oriented framework for molecular biomarker testing in kidney tumors. It integrates immunohistochemistry, fluorescence in situ hybridization, polymerase chain reaction-based methods, and next-generation sequencing with morphologic assessment. Particular attention is given to diagnostically relevant alterations involving *VHL*, *TFE3*, *TFEB*, *MET*, *FH*, *ELOC*, and *ALK*, genes encoding SDH subunits, and components of the mTOR signaling pathway, together with commonly used immunohistochemical surrogates. The prognostic and predictive roles of selected biomarkers, including their potential value in hereditary risk assessment and treatment selection, are also discussed. Practical limitations, such as limited biopsy material, intratumoral heterogeneity, and technical variability, are considered. **Conclusions**: Molecular biomarker testing is reshaping kidney tumor pathology by supporting an integrated diagnostic approach that complements, rather than replaces, careful morphologic evaluation. Its appropriate use can improve diagnostic accuracy, refine clinical stratification, and facilitate the recognition of emerging renal tumor entities with hereditary or therapeutic relevance. Future developments are expected to include methylation profiling, transcriptomic classifiers, liquid biopsy platforms, and artificial intelligence-assisted interpretation.

## Simple Summary

Kidney tumors include many distinct entities that may appear similar under the microscope but differ in their molecular features, clinical behavior, hereditary implications, and response to treatment. This review explains how molecular biomarkers can be integrated with routine microscopic examination and immunohistochemistry to improve tumor classification, identify newly recognized and hereditary renal tumor types, and support prognosis and treatment selection. It also highlights the practical limitations of current testing and discusses emerging approaches, including methylation profiling, transcriptomic classifiers, liquid biopsy, and artificial intelligence.

## 1. Introduction

Kidney tumors represent a biologically heterogeneous and increasingly complex family of neoplasms, with marked differences in morphology, genomic alterations, clinical behavior, and therapeutic responsiveness [1,2,3,4]. Contemporary renal tumor diagnostics encompass newly recognized molecularly defined entities, indolent neoplasms with distinctive genetic signatures, aggressive high-grade carcinomas, hereditary syndrome-associated lesions, and emerging provisional subtypes [1,3]. This evolution has transformed renal tumor diagnostics from a predominantly histologic exercise into an integrated discipline that combines morphology, immunophenotype, and molecular pathology for precise classification and clinical management. Although thorough morphologic assessment remains the cornerstone of renal tumor diagnosis, with architectural pattern and cytologic features providing the initial and often most informative clues, morphology alone is insufficient in a substantial subset of cases due to overlapping patterns across biologically distinct entities. In this context, molecular testing and immunohistochemistry (IHC) have become essential for resolving challenging differentials and confirming molecularly defined tumor types [5,6].

Reflecting this shift, the evolving WHO classification of renal tumors, including the forthcoming sixth-edition framework, recognizes an expanding spectrum of renal neoplasms in which molecular alterations are central to classification, including *TFE3*-rearranged renal cell carcinoma (RCC), *TFEB*-altered RCC, *ELOC*-mutated RCC, fumarate hydratase (FH)-deficient RCC, succinate dehydrogenase (SDH)-deficient RCC, *ALK*-rearranged RCC, and *SMARCB1*-deficient renal medullary carcinoma [1]. In parallel, immunohistochemical surrogates such as CA9, KRT7, AMACR, FH, SDHB, cathepsin K, and GPNMB have become highly valuable adjuncts in routine practice, while fluorescence in situ hybridization (FISH), polymerase chain reaction (PCR), and DNA- and RNA-based next-generation sequencing (NGS) enable direct detection of rearrangements, mutations, and copy number alterations [3,5,6,7,8,9].

Beyond diagnosis, biomarker testing increasingly influences patient management [10]. Molecular findings may refine prognostic stratification, predict response or resistance to antiangiogenic therapy, identify candidates for immune checkpoint blockade, and support selection of targeted therapies, while germline alterations may signal inherited predisposition with implications for affected individuals and their families [10,11]. Implementation must nonetheless be balanced against real-world challenges, including resource-limited laboratory settings, restricted biopsy tissue, intratumoral heterogeneity, pre-analytic variability, and context-dependent interpretation.

Recent reviews, including the work by Akgul et al., have provided focused guidance on the diagnosis of molecularly defined RCCs [5]. The present review extends this foundation by integrating molecularly defined and conventional renal tumor categories within a unified morphology-driven testing framework. In addition to diagnostic classification, it links immunohistochemical and molecular findings to therapeutic selection, hereditary risk assessment, tissue stewardship, assay-specific limitations, and emerging technologies. The proposed three-tier strategy and consolidated diagnostic summary are intended to translate this broader scope into a practical workflow for routine surgical pathology. For this narrative review, the literature was selected through targeted searches of PubMed and major pathology and oncology publications, with priority given to the WHO classification, consensus documents, clinically relevant studies, and recent original reports describing emerging renal tumor entities. The literature was critically synthesized according to diagnostic relevance, strength of clinical evidence, hereditary implications, and applicability to routine practice. Accordingly, we provide a practical and contemporary framework for molecular biomarker testing in kidney tumors, emphasizing diagnostic applications, therapeutic relevance, hereditary risk assessment, common pitfalls, and future directions in precision renal oncology.

## 2. Diagnostic and Predictive Biomarkers in Renal Tumors

The diagnostic evaluation of renal tumors increasingly relies on an integrated framework that combines morphology with a combination of biomarker assays [1,6]. Although many renal neoplasms retain characteristic histologic patterns, substantial overlap exists. In this context, biomarker testing is most helpful when applied in a morphology-driven and clinically targeted manner. The optimal diagnostic strategy therefore begins with recognition of the dominant morphologic phenotype, followed by selection of confirmatory markers tailored to the most relevant differential diagnoses. Although no single biomarker currently dictates management across all renal tumor subtypes, accumulating evidence supports a clinically meaningful framework in which pathway alterations, tumor microenvironment features, and lineage-specific molecular events can refine precision treatment approaches in renal neoplasia [10,12]. The principal morphologic, immunohistochemical, and molecular features of the renal tumor entities discussed below are consolidated in Table 1.

### 2.1. Renal Tumors with Clear Cytoplasm

Clear cell RCC (ccRCC) is the most common malignant renal epithelial neoplasm and is typically characterized by nests, alveolar structures, or acinar growth composed of cells with clear cytoplasm and a delicate vascular network [13]. Molecularly, ccRCC is driven by chromosome 3p loss and inactivation of the *VHL* pathway, leading to stabilization of hypoxia-inducible factors and downstream expression of hypoxia-responsive genes [14]. Immunohistochemically, diffuse membranous CA9 with a characteristic box-like staining pattern remains one of the most useful diagnostic markers, whereas KRT7 is absent or only focal in most conventional cases [6]. A practical initial panel for tumors with clear cytoplasm includes CA9, KRT7, and CD10. GATA3 may be added as a subtype-refining marker when clear cell papillary renal cell tumor is included in the differential diagnosis. Diffuse membranous CA9 with CD10 positivity and absent or only focal KRT7 strongly supports ccRCC, whereas diffuse KRT7, cup-like CA9 positivity, and GATA3 expression favor clear cell papillary renal cell tumor [15]. When morphology is unusual (papillary, cystic, or eosinophilic), CA9 and/or KRT7 pattern is nonclassic, or the tumor has leiomyomatous stroma, the differential expands to *TFE3*-rearranged RCC, *TFEB*-altered RCC, and *ELOC*-mutated RCC, prompting the use of second-line markers such as cathepsin K, GPNMB, and TFE3 IHC, with FISH or NGS reserved for confirmation [7,16].

Loss of functional VHL prevents oxygen-dependent degradation of HIF transcription factors and results in constitutive activation of HIF-regulated programs, including VEGFA-mediated angiogenesis, metabolic reprogramming, cell survival, and tumor progression. This pathway has several distinct biomarker implications that should not be conflated. VHL/HIF pathway activation is a defining biological feature of ccRCC, whereas HIF and VEGF expression have been investigated primarily as prognostic or treatment-associated biomarkers. HIF2α is also a therapeutically validated target, as demonstrated by the clinical activity of HIF2α inhibition; however, immunohistochemical expression of HIF or VEGF is not currently an established stand-alone predictive test for treatment selection. Importantly, nuclear and cytoplasmic HIF expression may convey different clinicopathological information because transcriptional activity depends principally on nuclear localization. 

Emerging data also suggest that alterations such as *PBRM1, BAP1*, *SETD2*, and *KDM5C* are associated with certain morphologic patterns and may offer useful diagnostic, biological or therapeutic insights in challenging cases, although they are not required for routine diagnosis [17,18,19,20]. In particular, *BAP1*-mutated ccRCC tends to display voluminous granular to densely eosinophilic cytoplasm with prominent eosinophilic hyaline globules, and high-grade nuclei, frequently accompanied by an adjacent conventional ccRCC component with an abrupt transition [19]. Immunohistochemically, these tumors typically show loss of nuclear BAP1 expression [19]. *BAP1* alterations have reproducible prognostic associations and are linked to aggressive behavior, including renal vein invasion, advanced pathological stage, and distant metastasis [19]. By contrast, their predictive significance remains unvalidated. Preclinical and early translational studies have suggested potential sensitivity to PARP inhibition and radiation and possible differences in response to mTOR inhibition [21,22]. These observations should be regarded as hypothesis-generating and do not currently support the use of BAP1 status as a stand-alone clinical treatment-selection biomarker.

### 2.2. Papillary Renal Neoplasms

Papillary renal neoplasms encompass a heterogeneous group. Traditional subdivision into type 1 and type 2 tumors has been replaced by distinct RCC subtypes, predominantly reflecting the biologic diversity of this category [1,23,24]. Molecularly, classic papillary RCC (formerly “type 1”) is characterized by gains of chromosomes 7 and 17, loss of chromosome Y, and activation of the *MET* signaling pathway [23,24]. *MET* alterations may take the form of activating point mutations, *MET* gene amplification, or copy number gains through chromosome 7 polysomy that effectively duplicate the wild-type *MET* allele [24,25,26]. Germline activating *MET* mutations underlie hereditary papillary RCC, in which affected individuals develop multifocal and bilateral papillary tumors, whereas somatic *MET* alterations drive a substantial subset of sporadic cases [27]. *MET* pathway activation provides the biologic rationale for *MET*-directed therapy, and selective MET inhibitors have shown activity in selected *MET*-altered papillary RCC cohorts [28]. However, therapeutic benefit varies according to the specific alteration and agent, and *MET* status should not be regarded as a universally validated predictive biomarker across all papillary RCCs. Within the *MET*-driven spectrum, biphasic squamoid alveolar RCC has emerged as a morphologically distinct subtype frequently harboring *MET* alterations [29]. It is characterized by a biphasic architecture composed of nests of larger squamoid cells surrounded by smaller alveolar cells, frequently demonstrating emperipolesis [29]. The proportion of the larger cell component is variable, and they typically show cyclin D1 expression [29].

Papillary adenoma (PA) is regarded as the precursor lesion of classic papillary RCC, and the two share a common molecular backbone: gains of chromosomes 7 and 17 and loss of chromosome Y are present in both, occur early in tumorigenesis, and likely represent the initiating cytogenetic events upon which subsequent oncogenic alterations are layered [30]. Integrated copy-number, miRNA, and whole-exome sequencing analyses have shown that PA and the major papillary RCCs share recurrently gained chromosomal regions, recurrent frameshift mutations in *KMT2C*, and pathogenic *DUSP5* variants [30]. Aberrant nuclear or cytoplasmic β-catenin accumulation is identifiable in the majority of PAs, consistent with WNT pathway activation as an early driver [30].

A recently recognized entity within the papillary tumor spectrum is papillary renal neoplasm with reverse polarity, characterized by eosinophilic cells with apically oriented low-grade nuclei and recurrent *KRAS* mutations [31,32]. Although *KRAS* mutations are typically actionable in other tumor types, the indolent course of these renal tumors generally obviates the need for targeted therapy.

The molecular landscape of former “type 2” papillary RCC is substantially more heterogeneous and no longer represents a unified entity. Many such tumors are now reclassified into molecularly defined tumors that carry distinct biology and management [1].

For practical work-up, an initial morphology-directed panel includes KRT7 and AMACR. GATA3 may be added as a subtype-refining marker when papillary renal neoplasm with reverse polarity is considered. Papillary RCC typically shows KRT7 and AMACR positivity. Papillary renal neoplasm with reverse polarity often expresses GATA3 [33]. When the morphology or initial IHC panel does not support classic papillary RCC, the differential should expand to FH-deficient RCC, TFE3-rearranged RCC, TFEB-altered RCC, or other molecularly defined entities.

### 2.3. Chromophobe RCC, Renal Oncocytoma, Low-Grade Oncocytic Tumor, and Other Low-Grade Oncocytic Neoplasms

The differential diagnosis between chromophobe RCC, renal oncocytoma, and the newly recognized low-grade oncocytic tumor (LOT) is one of the most common biomarker-driven challenges in renal tumor pathology. A practical initial panel includes KRT7 and KIT. Subtype-refining markers, including GATA3, L1CAM, SDHB, FH, cathepsin K, and GPNMB, may then be selected according to the morphology and the resulting differential diagnosis. Diffuse KRT7 with KIT positivity supports chromophobe RCC, which is also characterized by multiple chromosomal losses typically involving chromosomes 1, 2, 6, 10, 13, 17, and 21, whereas strong KIT with absent or only focal KRT7 supports oncocytoma [6,34].

Although chromophobe RCC has historically been considered to have a low mutational burden, recent studies have refined the molecular landscape. *MTOR*, *TSC1*, or *TSC2* mutations occur in approximately 10–17% of chromophobe RCCs, are enriched in the eosinophilic variant, and are independently associated with worse disease-specific survival and increased metastatic risk, raising the possibility of mTOR inhibitor benefit in this subset [35]. A small but clinically important subset of chromophobe RCC displays high-grade, nonsarcomatoid morphology, defined by nondegenerative nuclear atypia, increased cellularity, atypical mitoses, and necrosis. These tumors present at an advanced stage and harbor *RB1* mutations with corresponding loss of RB expression (or strong, diffuse P16 expression as a surrogate for functional RB loss) almost exclusively in the high-grade component, alongside chromosomal gains involving chromosomes 7, 16, 18, and 20 consistent with imbalanced chromosomal duplication [36,37]. Awareness of this subset is important because routine grading is not applied to chromophobe RCC, yet *RB1*-driven high-grade transformation is associated with markedly aggressive behavior [36,37].

KRT7-positive/KIT-negative tumors should raise consideration of LOT, now recognized as a distinct entity in the WHO classification [1]. LOT presents as a solitary, organ-confined tumor in older adults and behaves indolently [38]. Histologically, it shows compact nested to solid growth that transitions sharply into hypocellular edematous stromal areas containing loosely arranged tumor cells in cords, reticular structures, and individual cell patterns [38]. The cells display oncocytic cytoplasm with uniform, low-grade round-to-oval nuclei [38]. Importantly, LOT lacks the multiple chromosomal losses characteristic of chromophobe RCC, and aggregated NGS data from published series demonstrate strong involvement of the mTOR pathway, with *MTOR*, *TSC1*, and *TSC2* representing the most frequently mutated genes [38,39,40]. Additional non-canonical *mTOR*-activating alterations occur in *PIK3CA*, *STK11*, *PTEN*, and *NF2*, with *PIK3CA* mutations sometimes appearing as the sole driver event [40].

For low-grade oncocytic/eosinophilic renal neoplasms that do not fulfill the diagnostic criteria for oncocytoma, chromophobe RCC, LOT, EVT, ESC RCC, sporadic *FLCN*-mutated neoplasms, or syndrome-associated oncocytic tumors, the designation renal oncocytic neoplasm, NOS/NCF may be appropriate. This category should be restricted to morphologically low-risk tumors and should not be used in the presence of coagulative tumor necrosis, lymphovascular invasion, frequent or atypical mitoses, prominent nuclear atypia, sarcomatoid or rhabdoid features, or infiltrative/desmoplastic growth.

### 2.4. TFE3-Rearranged RCC and TFEB-Altered RCC

TFE3-rearranged RCC and TFEB-altered RCC are recognized as separate entities in the evolving WHO classification. TFEB-altered RCC encompasses TFEB-rearranged and TFEB-amplified tumors, which differ in their molecular background and clinical behavior. TFE3-rearranged RCC is a heterogeneous entity in which fusion partners may correlate with distinct morphologic patterns, immunophenotypic features, and clinical behavior [41,42]. Certain fusion partners appear to show relatively characteristic features. *MED15::TFE3* RCC is often associated with a predominantly multicystic architecture and has generally been linked to a more favorable outcome; *ASPSCR1::TFE3* RCC more frequently contains psammoma bodies and poor prognosis, whereas *SFPQ::TFE3* RCC has been reported to show melanotic pigment more often than others [41,42,43,44]. *MED15::TFE3* RCC may also demonstrate more frequent expression of melanocytic markers such as Melan-A [42]. Fusion partners may also have therapeutic relevance. In particular, *ASPSCR1::TFE3* RCC has been associated with a more angiogenic and immune-enriched tumor microenvironment compared with other TFE3 fusion partners, and emerging evidence suggests that combined immune checkpoint inhibitor and VEGFR tyrosine kinase inhibitor therapy may provide particular benefit in this subgroup [41].

In view of their marked morphologic heterogeneity, ancillary testing is necessary to establish the diagnosis. Nuclear TFE3 expression may be helpful as a screening tool but can be affected by fixation and laboratory protocols [7,45]. Both *TFE3-* and *TFEB*-rearranged RCCs have reduced pankeratin and EMA expression. Cathepsin K and GPNMB are valuable supportive markers [7,45]. Break-apart FISH remains a widely used confirmatory method, while RNA-based sequencing increasingly enables precise fusion partner identification and *TFE3*-rearranged tumors with cryptic inversions [7].

Recognition of TFE3-rearranged RCC and TFEB-altered RCC is clinically important because these tumors may occur in younger patients and may behave aggressively in adults; however, biomarker-directed therapeutic strategies remain investigational [46].

### 2.5. Fumarate Hydratase-Deficient Renal Cell Carcinoma

FH-deficient RCC is an uncommon entity that is defined by biallelic inactivation of the *FH* gene [47]. A majority of FH-deficient RCCs arise in the setting of hereditary leiomyomatosis and renal cell carcinoma (HLRCC) syndrome, whereas a significant minority arise sporadically through somatic-only *FH* inactivation [47,48]. Loss of FH activity drives intratumoral accumulation of fumarate, which acts as an oncometabolite, succinating cysteine residues on cellular proteins to generate 2-succino-cysteine (2SC), inhibiting α-ketoglutarate-dependent dioxygenases, and producing pseudohypoxic and hypermethylator phenotypes that promote aggressive tumor behavior [49].

Morphologically, FH-deficient RCC is heterogeneous, with papillary, tubulocystic, solid, cribriform, sieve-like, and collecting duct-like architectures often coexisting within the same tumor [47]. The most frequently emphasized cytologic clue is the presence of prominent eosinophilic macronucleoli with perinucleolar clearing reminiscent of viral inclusions [47]. The diagnosis rests on the combination of compatible morphology with characteristic IHC: loss of FH expression and/or aberrant 2SC positivity. FH loss is highly specific but incompletely sensitive because some functionally deficient tumors retain detectable FH expression, whereas 2SC is highly sensitive but may be less specific [48,50]. An oncocytic subtype of FH-deficient RCC is increasingly recognized, typically showing lower-grade cytologic features and morphologic resemblance to SDH-deficient RCC. These tumors appear to follow a less aggressive course than conventional FH-deficient RCC.

The recognition of FH-deficient RCC has direct therapeutic implications. Conventional VEGF tyrosine kinase inhibitor monotherapy and single-agent mTOR inhibitors produce limited responses, and single-agent immune checkpoint blockade has shown essentially no objective responses in retrospective metastatic series [51]. In contrast, combined antiangiogenic and EGFR-directed therapy has emerged as the historically favored regimen, with more recent prospective data on combined VEGFR tyrosine kinase inhibitor plus anti-PD-1 combinations reporting substantially higher objective response rates [52,53].

### 2.6. Succinate Dehydrogenase-Deficient Renal Cell Carcinoma

SDH-deficient RCC is among the rarest renal entities, accounting for roughly 0.05–0.2% of all RCCs [54]. It is defined by biallelic inactivation of one of the genes encoding the four subunits of the mitochondrial complex II enzyme (*SDHA*, *SDHB*, *SDHC*, *SDHD*), with *SDHB* mutations representing the majority of cases [54,55]. Loss of SDH activity causes accumulation of succinate, a second oncometabolite that inhibits α-ketoglutarate-dependent dioxygenases and drives pseudohypoxic signaling and aberrant DNA and histone methylation [56,57]. Most cases arise on a germline background, and patients are generally diagnosed at younger ages than those with sporadic RCC [54].

Histologically, SDH-deficient RCC has a distinctive appearance characterized by solid or nested growth of cuboidal cells with bubbly, flocculent, vacuolated, or pale eosinophilic cytoplasm [54]. Most tumors display low-grade nuclei and lack significant pleomorphism, although a small subset shows high-grade nuclei, sarcomatoid differentiation, or coagulative necrosis associated with worse outcome [54,58,59]. The diagnostic immunohistochemical hallmark is complete loss of SDHB expression in tumor cells regardless of which specific SDH subunit harbors the underlying mutation [54,59]. Also, GATA3 positivity with no KIT and KRT7 expression helps to identify these tumors [60]. Identification of SDH-deficient RCC essentially mandates referral for genetic counseling and germline testing.

Most SDH-deficient RCCs are low-grade and clinically indolent, and partial nephrectomy with negative margins is generally curative; the presence of adverse pathologic features, high-grade nuclei, coagulative necrosis, or sarcomatoid differentiation identifies a small subset with substantially higher metastatic potential that warrants closer follow-up [54]. Systemic therapy evidence in metastatic SDH-deficient RCC is essentially limited to anecdotal case reports, and no class of agents has been prospectively validated in this entity; selection is typically extrapolated from non-clear cell RCC management, with VEGF receptor tyrosine kinase inhibitors most commonly employed [61].

### 2.7. Other Molecularly Defined Renal Cell Carcinomas

Beyond the TFE3-rearranged or TFEB-altered renal tumors, FH-deficient RCC, and SDH-deficient RCC, the 5th edition WHO classification formally recognizes three additional renal cell carcinomas defined primarily by their molecular alterations rather than by morphology alone: *ELOC*-mutated RCC, *ALK*-rearranged RCC, and SMARCB1-deficient renal medullary carcinoma [1]. These tumors are individually rare but collectively important because each carries distinctive diagnostic features and, in selected cases, direct therapeutic implications that diverge from those of conventional RCCs.

*ELOC*-mutated (formerly *TCEB1*-mutated) RCC is defined by hotspot somatic missense mutations in *ELOC* accompanied by monosomy 8 (encompassing the *ELOC* locus on 8q21) [16,62]. The resulting elongin C dysfunction disrupts the *VHL*-elongin complex and produces constitutive HIF stabilization [62,63]. Morphologically, *ELOC*-mutated RCC shows clear cells arranged in nests or tubules surrounded by prominent smooth muscle stroma [16,64]. These tumors are diffusely positive for CA9 and KRT7 [64]. Diagnosis is supported by characteristic morphology and immunoprofile, with confirmation by targeted *ELOC* sequencing [64,65]. Behavior is generally indolent, and most cases are managed surgically; given the HIF-driven biology, antiangiogenic VEGF-targeted therapy has a mechanistic rationale; however, there are no prospective clinical data demonstrating entity-specific benefit [16].

*ALK*-rearranged RCC is a rare molecularly defined RCC characterized by structural alterations resulting in *ALK* gene fusions. Reported fusion partners include *EML4*, *TPM3*, *VCL*, *TPM1*, *SLIT1*, *STRN*, *HOOK1*, *KIF5B*, *CLIP1*, *KIAA1217*, *PLEKHA7*, *NUMA1*, and *ARNT* [66,67]. Morphology is highly heterogeneous and may include papillary, tubular, tubulocystic, cribriform, solid, trabecular, or corded architectures, frequently with eosinophilic cytoplasm and extracellular mucin. ALK immunohistochemistry typically shows strong and diffuse cytoplasmic and/or membranous expression and is useful as a screening tool. However, definitive diagnosis requires demonstration of an *ALK* rearrangement or fusion by break-apart FISH, next-generation sequencing, or RT-PCR. TFE3 immunoreactivity may occur despite the absence of a *TFE3* rearrangement and represents a potential diagnostic pitfall. Although many localized tumors follow an indolent course, metastatic progression is well documented. ALK inhibitors have produced clinically meaningful responses in selected metastatic cases, but the available predictive evidence remains limited and heterogeneous [68,69,70].

SMARCB1-deficient renal medullary carcinoma (RMC) is a highly aggressive carcinoma arising almost exclusively in young patients with sickle cell trait or, less commonly, sickle cell disease or hemoglobin SC disease [71]. It is characterized by biallelic inactivation of *SMARCB1* (*INI1*), a core subunit of the SWI/SNF chromatin remodeling complex, most often through deletion or rearrangement rather than point mutation [71]. Tumors arise in the renal medulla and display infiltrative growth with reticular, cribriform, or solid architecture, high-grade rhabdoid cytomorphology, and a brisk neutrophilic and lymphocytic infiltrate [71]. The diagnostic hallmark is complete loss of INI1 nuclear expression by immunohistochemistry [71,72]. OCT3/4 expression has also been described and may be a useful adjunct [73]. The differential diagnosis includes urothelial carcinoma with rhabdoid features, collecting duct carcinoma, and SMARCB1-deficient sarcomatoid neoplasms, all of which require careful integration of morphology, clinical context, and immunohistochemistry. Therapeutically, they are resistant to conventional cytotoxic regimens used for clear cell RCC and to single-agent VEGF-targeted therapy [74]. Platinum-based combination chemotherapy, particularly cisplatin, paclitaxel, and gemcitabine, remains the backbone of systemic management [74,75]. The addition of the proteasome inhibitor bortezomib to platinum-based chemotherapy has been associated with complete responses in two reported pediatric cases of renal medullary carcinoma; however, the evidence remains limited to individual case reports, and the efficacy of this approach requires further investigation [76].

### 2.8. Other Renal Tumors with Distinct Molecular Alterations

A growing number of renal neoplasms display reproducible morphologic, immunophenotypic, and molecular features, although their current classification status and level of supporting evidence vary. Several tumors that were initially described as emerging or provisional categories have now acquired defined diagnostic criteria and recognition as distinct entities in the evolving WHO classification, whereas other molecularly characterized renal neoplasms remain incompletely defined. These categories should therefore not be considered equivalent with respect to classification status or strength of supporting evidence. Awareness of these tumors is increasingly important as targeted sequencing becomes more widely available.

Biphasic hyalinizing psammomatous RCC is a recently characterized renal neoplasm composed of a biphasic population of larger and smaller epithelial cells, with the smaller cells commonly clustering around basement membrane material to produce pseudorosette-like or glomeruloid structures. Hyalinized or sclerotic stroma and psammomatous calcifications are frequent [77]. The tumors usually express PAX8, vimentin, and AMACR, whereas KRT7 expression is variable. Loss of merlin, the protein product of *NF2*, is a useful immunohistochemical surrogate for *NF2* inactivation and has shown approximately 92% sensitivity and 94% specificity for tumors within the classic BHP RCC spectrum [78]. Molecularly, these tumors demonstrate biallelic *NF2* inactivation or, less commonly, *NF2* promoter methylation. Biphasic hyalinizing psammomatous RCC is recognized as a distinct renal tumor entity characterized by its defining biphasic morphology together with *NF2* inactivation and/or loss of merlin expression. Importantly, *NF2* inactivation alone is not sufficient for this diagnosis, as *NF2* alterations may also occur in other renal tumor types. Renal tumors showing *NF2* inactivation but lacking the characteristic morphologic features of BHP RCC should therefore not be classified as BHP RCC solely on molecular grounds. Available clinical data remain limited, although metastatic and clinically aggressive behavior has been documented in a subset of cases [79,80].

Follicular (thyroid-like) RCC is a molecularly defined renal epithelial neoplasm characterized by *EWSR1::PATZ1* fusion [81,82]. Histologically, it is composed of closely packed, variably sized tubules resembling thyroid follicles and containing eosinophilic colloid-like material. Tumor cells consistently express PAX8, KRT7, and cyclin D1, whereas TTF1, thyroglobulin, and WT1 are negative, supporting renal origin and helping exclude metastatic thyroid carcinoma. Demonstration of *EWSR1::PATZ1* fusion or *EWSR1* rearrangement is essential for diagnosis in the appropriate morphologic and clinical context. Break-apart FISH may fail to detect some *EWSR1::PATZ1* fusions because the two genes are located in close proximity on chromosome 22 and the fusion may arise through paracentric inversion; NGS-based fusion detection is therefore preferable when suspicion remains high despite negative FISH [82,83]. Although available clinical experience remains limited, regional and distant metastases have been documented, particularly in tumors showing sarcomatoid transformation [83].

**Figure 1:**
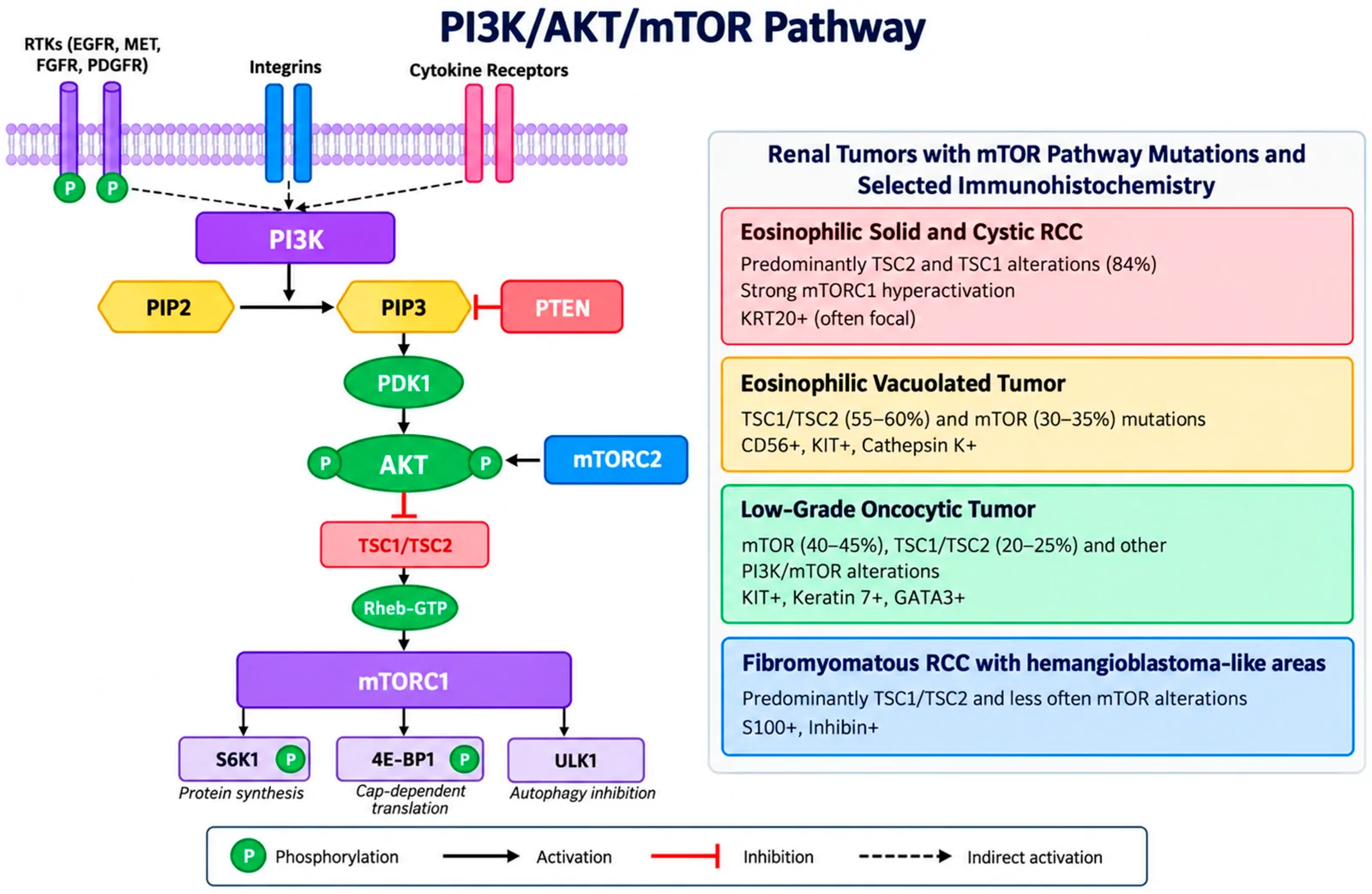
PI3K/AKT/mTOR signaling pathway and representative renal neoplasms characterized by mTOR pathway alterations. Schematic overview of the canonical PI3K/AKT/mTOR signaling cascade and its relevance in renal tumorigenesis. Activation of upstream RTKs, integrins, and cytokine receptors stimulates PI3K, leading to conversion of PIP2 to PIP3, recruitment of PDK1, and activation of AKT. PTEN acts as a key negative regulator by antagonizing PIP3 accumulation. Activated AKT suppresses the TSC1/TSC2 complex, promoting accumulation of active RHEB-GTP and subsequent activation of mTORC1, while mTORC2 contributes to AKT activation through feedback phosphorylation. Downstream mTORC1 signaling promotes protein synthesis and cellular growth through S6K1 and 4E-BP1 while suppressing autophagy via ULK1 inhibition. Representative renal neoplasms with recurrent mTOR pathway alterations are shown, including eosinophilic solid and cystic RCC, eosinophilic vacuolated tumor, low-grade oncocytic tumor, and fibromyomatous RCC with hemangioblastoma-like areas, together with selected characteristic immunohistochemical features. The relative frequencies of TSC1, TSC2, and MTOR alterations vary among tumor types and published cohorts and should not be interpreted as fixed entity-specific percentages. **Abbreviations**: AKT: protein kinase B, Cathepsin K: cathepsin K, ESC RCC: eosinophilic solid and cystic renal cell carcinoma, EVT: eosinophilic vacuolated tumor, GATA3: GATA binding protein 3, KIT: KIT proto-oncogene receptor tyrosine kinase (CD117), KRT20: cytokeratin 20, LOT: low-grade oncocytic tumor, mTOR: mechanistic target of rapamycin, mTORC1: mechanistic target of rapamycin complex 1, mTORC2: mechanistic target of rapamycin complex 2, PDK1: phosphoinositide-dependent kinase 1, PI3K: phosphoinositide 3-kinase, PIP2: phosphatidylinositol-4,5-bisphosphate, PIP3: phosphatidylinositol-3,4,5-trisphosphate, PTEN: phosphatase and tensin homolog, RCC: renal cell carcinoma, RHEB: Ras homolog enriched in brain, RTK: receptor tyrosine kinase, S6K1: ribosomal protein S6 kinase beta-1, TSC1/TSC2: tuberous sclerosis complex 1/2, ULK1: unc-51-like kinase 1.

The mTOR pathway-altered renal tumor spectrum has expanded substantially and encompasses several morphologically distinct neoplasms with recurrent alterations involving *TSC1*, *TSC2*, *MTOR*, *PIK3CA*, or *PTEN* (Figure 1) [39].

Eosinophilic solid and cystic RCC (ESC-RCC) is defined by an admixture of solid eosinophilic sheets and macrocysts lined by hobnail cells, with abundant granular cytoplasm and basophilic cytoplasmic stippling [84,85]. Tumor cells frequently express CK20 in a focal patchy pattern [84,85]. ESC-RCC is the most strongly *TSC1/2*-driven entity in the spectrum, with biallelic *TSC2* alterations identifiable in over 80% of cases [39]. The female predominance, sporadic occurrence in adults, and indolent course are now well established [84,85].

Eosinophilic vacuolated tumor (EVT) is characterized by solid, nested, acinar, or trabecular growth of large eosinophilic cells with prominent nucleoli and conspicuous intracytoplasmic vacuoles [86,87]. EVT shows a reproducible immunophenotype, with expression of CD117, cathepsin K, CD10, cytokeratin 8/18, and frequently GPNMB, whereas KRT7 is negative or limited to focal or patchy staining. Vimentin, HMB45, and Melan-A are typically negative [8,86,87]. Molecular alterations involve *TSC1, TSC2*, or activating *MTOR* mutations, with reported frequencies varying among published cohorts because of small sample sizes and differences in case selection and molecular platforms [39,86,87]. These alterations converge on mTORC1 pathway activation. Although most EVTs behave indolently, rare metastatic examples have now been documented [88].

Hemangioblastoma-like morphology has been documented within TSC/mTOR-altered fibromyomatous RCC and is characterized by an arborizing capillary network and stromal-cell–like tumor cells with pale, vacuolated, or eosinophilic cytoplasm [89]. These hemangioblastoma-like areas typically express inhibin-α, S100, and GPNMB but lack KRT7, whereas the conventional fibromyomatous RCC component is KRT7-positive and -negative for inhibin-α and S100. Recognition of this biphasic immunophenotype supports interpretation of hemangioblastoma-like areas as part of the fibromyomatous RCC spectrum rather than as a separate renal tumor entity [89].

Folliculin-mutated renal tumors are characterized by biallelic inactivation of *FLCN*, either in association with Birt–Hogg–Dubé syndrome or, less commonly, through purely somatic alterations [90,91,92,93]. Conventional tumors commonly contain an admixture of eosinophilic and clear cells, sometimes forming a mosaic or checkerboard pattern, whereas non-conventional tumors may show papillary, tubulopapillary, microcystic, tubulocystic, or other heterogeneous architectures. Immunohistochemically, these tumors usually show diffuse PAX8 and GPNMB expression, with mosaic or variable staining for KRT7, GATA3, CD117, and L1CAM [90,91,92,93]. Because morphologic and immunophenotypic overlap with TSC/MTOR-altered, TFE3-rearranged, and TFEB-altered renal tumors can be substantial, demonstration of biallelic FLCN inactivation may be required for definitive classification. Importantly, an apparently sporadic *FLCN*-mutated renal neoplasm may represent either a tumor with purely somatic biallelic alterations or the first manifestation of previously unrecognized Birt–Hogg–Dubé syndrome. Detection of pathogenic *FLCN* alterations, particularly in patients with multifocal or bilateral tumors or compatible personal or family history, should therefore prompt genetic counseling and consideration of confirmatory germline testing [90,91,92,93].

## 3. Therapeutic Implications and an Integrated Testing Strategy

Beyond classification, several molecular alterations in renal tumors have therapeutic relevance, although the strength of evidence differs substantially among biomarkers and tumor entities (Table 2). In ccRCC, *VHL* pathway inactivation provides the biological basis for established VEGF-targeted therapies and HIF2α inhibition [94,95,96]. However, *VHL* status and HIF/VEGF immunohistochemical expression are not routinely used as stand-alone predictive tests. Transcriptomic profiling suggests that angiogenic and immune-inflamed phenotypes may be associated with differential therapeutic outcomes, but no single biomarker reliably predicts response to immune checkpoint blockade in RCC [10]. In papillary RCC, selected *MET* alterations may identify tumors with sensitivity to MET-directed therapy, although the evidence varies by alteration and agent [97]. For rare tumors harboring *ALK* rearrangements or *TSC1/TSC2/MTOR* alterations, therapeutic evidence is based mainly on small series, case reports, retrospective observations, or biologic rationale rather than prospective validation [39,69,70,98]. Accordingly, the identification of a potentially actionable alteration should not automatically be interpreted as evidence of a clinically validated predictive biomarker. Intratumoral heterogeneity and clonal evolution remain additional limitations, and future predictive models will likely require integration of genomic, transcriptomic, and microenvironmental data [10,99].

In this context, a coherent diagnostic strategy must integrate morphology, immunohistochemistry, and molecular testing in a stepwise, hypothesis-driven manner rather than relying on indiscriminate panel ordering. A practical diagnostic framework can be conceptualized in three tiers. Tier 1 includes morphology-guided, lineage- and phenotype-defining immunostains (e.g., PAX8, CA9, KRT7, AMACR, CD10, KIT). Tier 2 incorporates subtype-refining markers (e.g., FH, 2SC, SDHB, TFE3, cathepsin K, GATA3, GPNMB, L1CAM, INI1), selected according to the narrowed differential diagnosis. Tier 3 comprises confirmatory molecular assays, employed when classification remains uncertain or when molecular findings may influence therapeutic decisions or hereditary risk assessment.

Escalation to molecular testing is most appropriate in morphologically ambiguous tumors following initial immunohistochemical work-up, in cases suspicious for molecularly defined entities, when morphology and immunophenotype are discordant, in tumors occurring in younger patients, in bilateral or multifocal neoplasms, or when hereditary syndromes are a consideration. Tissue stewardship is critical, particularly in core biopsies, where excessive reflex testing may deplete material before clinically meaningful analyses are performed.

**Figure 2:**
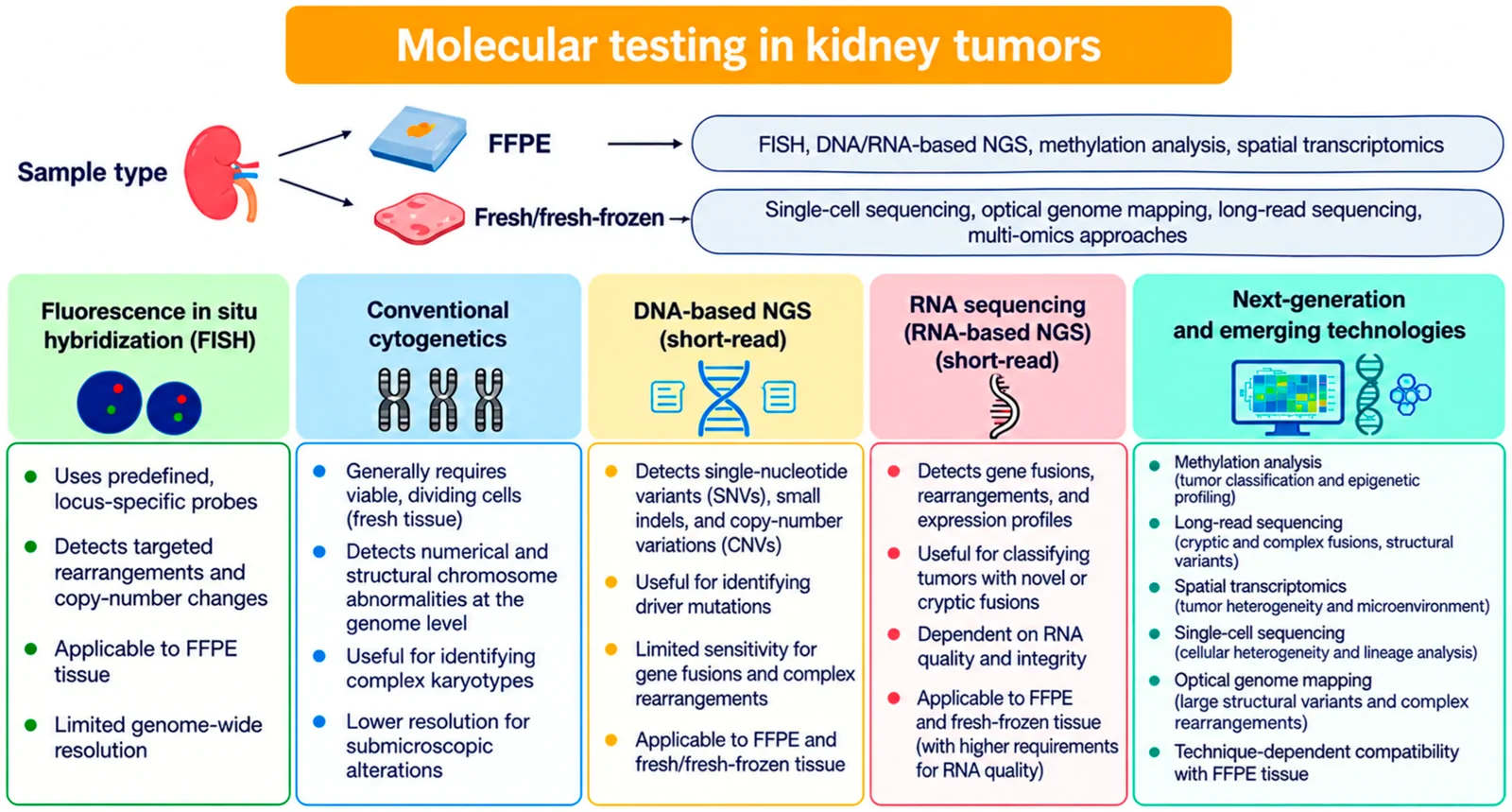
Overview of molecular testing platforms used in kidney tumors. This figure complements the morphology-driven diagnostic strategy described in the text by summarizing the molecular platforms available after histomorphologic and immunohistochemical assessment. FFPE tissue is suitable for FISH, DNA- and RNA-based next-generation sequencing, methylation profiling, and selected spatial transcriptomic approaches, whereas fresh-frozen tissue enables advanced applications, including single-cell sequencing, optical genome mapping, and long-read sequencing. The strengths and major limitations of each platform are summarized. **Abbreviations**: CNV, copy-number variant; DNA, deoxyribonucleic acid; FFPE, formalin-fixed, paraffin-embedded; FISH, fluorescence in situ hybridization; NGS, next-generation sequencing; RNA, ribonucleic acid; SNV, single-nucleotide variant.

Selection of the appropriate molecular platform requires balancing resolution, genomic breadth, and clinical necessity (Figure 2). FISH is well suited for targeted detection of predefined rearrangements, including *TFE3* and *TFEB*, but is inherently limited by probe design and may miss cryptic rearrangements [7]. Conventional cytogenetics, in contrast, provides genome-wide chromosomal information but has substantially lower resolution and requires viable dividing cells; it should therefore be considered separately from probe-based FISH assays. DNA-based short-read NGS is optimal for detecting single-nucleotide variants and small insertions/deletions, with limited capability for identifying large structural variants or balanced translocations [100]. In contrast, RNA-based NGS is particularly effective for detecting gene fusions, including novel chimeric transcripts involving known drivers; however, its performance is highly dependent on RNA quality and expression levels, which may be suboptimal in archival specimens [101,102]. Importantly, somatic NGS findings may also raise suspicion for germline predisposition, particularly when high variant allele frequencies or syndrome-associated alterations are observed, although confirmatory germline testing remains essential [103,104]. Figure 2 also summarizes emerging platforms (including methylation profiling, spatial transcriptomics, single-cell sequencing, optical genome mapping, and long-read sequencing) that remain primarily investigational in renal tumor pathology and are discussed further in Section 5.

### Hereditary Risk Assessment and Germline Testing

Molecular biomarker testing in renal tumors also plays an important role in identifying patients who may harbor a hereditary cancer predisposition. Features that should prompt consideration of genetic counseling and germline testing include early age at diagnosis, bilateral or multifocal renal tumors, a suggestive personal or family history, multiple oncocytic neoplasms, and the diagnosis of a tumor type strongly associated with an inherited syndrome [11,105]. In this setting, pathology may provide the first indication of an otherwise unrecognized hereditary disorder.

Several tumor findings warrant particular attention. FH-deficient RCC and SDH-deficient RCC should generally trigger genetic counseling and germline evaluation because of their strong associations with germline alterations in *FH* and the *SDHx* genes, respectively [47,54]. Likewise, multiple clear cell RCCs may raise suspicion for von Hippel–Lindau syndrome, whereas bilateral or multifocal papillary RCCs can suggest hereditary papillary RCC associated with germline *MET* alterations [106,107]. Multiple oncocytic or hybrid oncocytic/chromophobe tumors should prompt consideration of Birt–Hogg–Dubé syndrome, particularly when accompanied by renal oncocytosis or compatible extra-renal manifestations [108,109,110]. Renal tumors occurring in the setting of tuberous sclerosis complex may similarly show alterations involving *TSC1*, *TSC2*, or *mTOR* [39,111].

Immunohistochemical biomarkers can serve as practical screening tools in this context. Loss of FH expression and/or diffuse 2SC positivity supports FH deficiency, whereas loss of SDHB expression identifies SDH-deficient neoplasia regardless of the affected SDH subunit [48,54,112]. However, immunohistochemistry does not distinguish germline from purely somatic inactivation. Similarly, detection of a pathogenic variant in a syndrome-associated gene by tumor-only sequencing should be regarded as a potential indication for germline evaluation rather than as proof of hereditary disease. Variant allele frequency, tumor purity, copy-number status, and the specific gene involved should be considered when interpreting such findings [103,104].

Accordingly, suspected hereditary findings should be clearly communicated in the pathology report, with a recommendation for genetic counseling and confirmatory testing using a non-neoplastic specimen. Conversely, a negative tumor-based assay does not completely exclude a germline predisposition when the clinical or pathologic suspicion remains high [113]. Integrating morphology, immunohistochemistry, somatic molecular findings, and clinical context therefore allows biomarker testing to function not only as a diagnostic tool but also as a gateway to appropriate hereditary risk assessment.

## 4. Pitfalls and Limitations

Although molecular biomarker testing has substantially advanced renal tumor diagnostics, its real-world value depends on specimen quality, pre-analytic conditions, biologic context, and correlation with morphology. Biomarkers should not be interpreted as stand-alone truth tests, and overreliance on isolated marker results may lead to diagnostic overcalling, under-recognition of unusual entities, or inappropriate escalation to costly molecular studies.

The most common immunohistochemical pitfalls follow this same principle and each marker behaves predictably within a defined morphologic context. KRT7 supports the diagnosis of papillary RCC, classic chromophobe RCC, and LOT but its distribution and intensity must be interpreted alongside morphology [6]. For example, the eosinophilic chromophobe RCC may show focal or even absent KRT7 staining. AMACR is frequently positive in papillary RCC but variable expression has been documented in several molecularly defined entities and *BAP1*-mutated ccRCCs [19]. GATA3 expression can be seen in both LOT and SDH-deficient RCC [60]. Technical performance is similarly vulnerable to pre-analytic variability, with fixation quality, ischemic time, antibody clone selection, and platform differences all influencing results. Nuclear markers such as TFE3 are particularly prone to both false-positive staining and false-negative staining [114]. FISH-based confirmation has its own blind spots. Break-apart TFE3 FISH probes can fail to detect rearrangements that arise from small paracentric inversions on the X chromosome, in which the two halves of the split probe remain too close to be resolved as separate signals [7,115]. RNA-based NGS is more reliable in this setting and should be considered when morphology and immunophenotype strongly suggest a TFE3-rearranged tumor despite negative FISH. NGS assays more broadly may fail because of low tumor fraction, nucleic acid degradation, or insufficient yield, and somatic NGS in particular carries a non-trivial risk of both false-positive and false-negative calls owing to limited panel coverage and the inability to detect non-targeted or cryptic fusions [100,102]. A technically negative molecular result should therefore be interpreted cautiously whenever clinicopathologic suspicion remains high.

Two further considerations apply broadly. First, RCC is biologically heterogeneous at both morphologic and genomic levels, so a single core biopsy may not capture clinically relevant grade, sarcomatoid transformation, immune microenvironment, or subclonal alterations, a limitation especially relevant when biomarkers are used for therapeutic prediction or when negative results are used to exclude a targeted alteration [99]. Second, detection of a pathogenic alteration on tumor-only sequencing does not automatically establish hereditary disease, and a negative somatic result does not exclude a germline syndrome [103,104]. Definitive distinction requires matched germline testing with appropriate counseling [113].

Finally, the diagnostic landscape itself is moving. Renal tumor taxonomy continues to evolve, with entities increasingly subdivided by molecular features. Practical considerations also shape what is achievable in routine practice: access to advanced molecular testing remains uneven across institutions and health systems, and cost, turnaround time, and reimbursement constraints all influence test utilization. Throughout, morphology remains the foundation on which biomarker and molecular data must be interpreted in a morphology-driven differential.

## 5. Future Directions

The next phase of renal tumor pathology will be defined by deeper biologic profiling, more dynamic biomarkers, and tighter integration between pathology and precision oncology. Future classification systems are expected to incorporate multilayered data that better capture tumor lineage, evolution, therapeutic vulnerability, and microenvironmental complexity, with biomarkers functioning as decision-support tools across diagnosis, prognosis, treatment selection, and disease monitoring rather than as static labels. The relationship between these emerging technologies, specimen requirements, and currently established molecular assays is summarized in Figure 2.

Gene expression profiling is already revealing biologically meaningful subgroups within morphologically similar renal tumors, and future classifiers will likely integrate genomic alterations with transcriptomic, proteomic, and metabolomic signatures to distinguish aggressive from indolent disease more accurately than morphology alone [116]. DNA methylation profiling, which has transformed central nervous system tumor diagnostics, may offer similar value in renal neoplasia, particularly for unclassified tumors, oncocytic neoplasms with overlapping morphology, and metabolically driven entities in which oncometabolite accumulation directly influences chromatin regulation [56,117].

Newer sequencing technologies are also reshaping what can be resolved. As summarized in Figure 2, optical genome mapping provides a genome-wide view of structural architecture and excels in detecting large-scale, cryptic translocations and inversions that define certain RCC subtypes, although it does not yield nucleotide-level data [118]. Long-read sequencing resolves complex breakpoints and can simultaneously capture DNA methylation profiles [119]. In parallel, liquid biopsy approaches may eventually assist in minimal residual disease detection, treatment response monitoring, and tracking clonal evolution [120].

Experimental studies have implicated Hedgehog/SHH–SMO–GLI signaling in ccRCC biology. Expression-based analyses have suggested an association between SHH/GLI1 signaling and the VHL–HIF–VEGFA axis, although the direction and prognostic significance of GLI1 expression remain inconsistent across studies [121]. More recent experimental evidence indicates that primary cilia-dependent Hedgehog signaling may promote tumor growth and metastatic progression, particularly in VHL-wild-type ccRCC, and that pathway inhibition can induce autophagic cell death in preclinical models [122]. Nevertheless, Hedgehog signaling remains an investigational research area and is not currently established as a routine diagnostic, prognostic, or predictive biomarker in RCC.

Artificial intelligence and spatial biology are expected to complement rather than replace molecular pathology. Computational analysis of whole-slide images may help predict molecular alterations, quantify intratumoral heterogeneity, and triage cases for reflex biomarker testing, while spatial transcriptomics, multiplex imaging, and single-cell analysis can clarify why morphologically similar tumors behave differently and how stromal and immune compartments shape treatment response [123]. Taken together, these developments suggest that future renal tumor reports will increasingly include therapeutic vulnerabilities and hereditary risk flags.

## 6. Conclusions

Molecular biomarkers complement, rather than replace, expert morphologic assessment in kidney tumor pathology. Their principal clinical value lies in resolving challenging differential diagnoses, confirming molecularly defined entities, identifying hereditary risk, and, in selected settings, informing prognosis and treatment. Because assay performance is influenced by specimen quality, intratumoral heterogeneity, and technical limitations, testing should remain morphology-driven and supported by careful tissue stewardship. As transcriptomic, liquid biopsy, spatial, and artificial intelligence-assisted approaches mature, integrated interpretation of morphologic, immunophenotypic, and molecular findings will become increasingly important for accurate classification and personalized patient management.

## Figures and Tables

**Table 1:** Practical morphology-driven diagnostic framework for renal tumor entities, including key immunohistochemical profiles, defining or supportive molecular alterations, and principal clinical implications.

Entity	Key Morphologic Clues	Key Immunohistochemical Profile	Defining or Supportive Molecular Finding	Principal Diagnostic/Clinical Relevance
Clear cell RCC	Nested, alveolar, or acinar growth; clear cytoplasm; delicate vasculature	CA9 diffuse box-like; CD10 positive; KRT7 negative or focal	Chromosome 3p loss; biallelic *VHL* inactivation	HIF/VEGF pathway biology; selected cases may harbor prognostically relevant *BAP1*, *PBRM1*, or *SETD2* alterations
Clear cell papillary renal cell tumor	Tubulopapillary growth; clear cells; apically oriented nuclei	KRT7 diffuse; CA9 cup-like; GATA3 positive	Lacks canonical *VHL* inactivation/3p loss	Indolent tumor; distinction from clear cell RCC
Papillary RCC	Papillary/tubulopapillary growth; basophilic cells; foamy macrophages; hemosiderin	AMACR and CD10 positive; KRT7 usually positive; FH retained	Gains of chromosomes 7 and 17; loss of Y; *MET* alterations in a subset	Distinction from molecularly defined papillary-patterned tumors; potential relevance of *MET*-directed therapy
Papillary renal neoplasm with reverse polarity	Papillae lined by eosinophilic cells with apically oriented low-grade nuclei	GATA3 positive; KRT7 and AMACR frequently positive	Recurrent *KRAS* mutation	Indolent entity
Chromophobe RCC	Solid or nested growth; pale/eosinophilic cells; raisinoid nuclei; perinuclear halos	KIT positive; KRT7 usually diffuse	Multiple chromosomal losses; *MTOR/TSC1/TSC2* alterations in a subset	Recognition of high-grade *RB1*-altered tumors with aggressive potential
Renal oncocytoma	Nested or tubular oncocytic tumor; central scar may be present	KIT positive; KRT7 negative or limited to scattered cells	Characteristic chromosomal alterations, including loss of chromosome 1 in a subset	Distinction from chromophobe RCC and other oncocytic tumors
Low-grade oncocytic tumor	Solid/nested oncocytic areas transitioning to edematous stroma	KRT7 diffuse; KIT negative; GATA3 positive	*MTOR*, *TSC1*, *TSC2*, or other mTOR-pathway alterations	Generally indolent
TFE3-Rearranged RCC	Heterogeneous papillary, nested, cystic, or solid morphology; psammoma bodies in some fusion types	TFE3 nuclear expression; cathepsin K and GPNMB variably positive; keratin/EMA often reduced	*TFE3* fusion demonstrated by FISH or RNA-based NGS	FISH may be negative in cryptic inversions; fusion partner may have biologic relevance
TFEB-altered RCC	Biphasic or nested morphology; basement membrane material; melanocytic phenotype in some cases	Cathepsin K and GPNMB commonly positive; melanocytic markers variable	*TFEB* rearrangement or amplification	Rearranged and amplified tumors may have different behavior
FH-deficient RCC	Mixed papillary, tubulocystic, cribriform, or solid growth; prominent eosinophilic nucleoli with perinucleolar clearing	FH loss and/or diffuse 2SC positivity	Biallelic *FH* inactivation	Genetic counseling and germline testing are recommended; usually, but not uniformly, aggressive clinical behavior; oncocytic subtype may show a less aggressive course
SDH-deficient RCC	Nested or solid growth; flocculent eosinophilic cytoplasm; cytoplasmic vacuoles/inclusions	SDHB loss; GATA3 and L1CAM frequently positive; KIT and KRT7 usually negative	Biallelic alteration of an *SDHx* gene	Strong association with hereditary predisposition
ELOC-mutated RCC	Clear cells in nests or tubules with fibromyomatous stroma	CA9 and KRT7 positive	Biallelic *ELOC* inactivation, frequently with chromosome 8 loss	Usually indolent; molecular confirmation required
ALK-rearranged RCC	Variable papillary, tubular, mucinous, solid, or medullary-like morphology	*ALK* strong and diffuse; PAX8 positive; KRT7 frequently positive	*ALK* rearrangement/fusion demonstrated by break-apart FISH, RNA-based NGS, or RT-PCR	Molecular confirmation is essential; clinically meaningful responses to ALK inhibitors have been reported in selected metastatic cases
SMARCB1-deficient renal medullary carcinoma	Medullary location; reticular/cribriform growth; rhabdoid cytology; inflammatory infiltrate	Complete INI1 loss; OCT3/4 may be positive	Biallelic *SMARCB1* inactivation	Strong association with sickle hemoglobinopathy; highly aggressive
Biphasic hyalinizing psammomatous RCC	Biphasic large and small cells; pseudorosettes or glomeruloid structures; hyalinized stroma; psammoma bodies	*NF2* inactivation; loss of merlin expression as an immunohistochemical surrogate	Biallelic *NF2* inactivation or *NF2* promoter methylation	Distinct morphologically defined entity; *NF2* inactivation alone is insufficient for diagnosis because it may occur in other renal tumor types; metastatic behavior has been documented in a subset of cases.
Follicular (thyroid-like) RCC	Closely packed thyroid-like follicles containing eosinophilic colloid-like material	PAX8, KRT7, and cyclin D1 positive; TTF1, thyroglobulin, and WT1 negative	*EWSR1::PATZ1* fusion; FISH may have limited sensitivity because of the close genomic proximity of *EWSR1* and *PATZ1*	Exclusion of metastatic thyroid carcinoma; demonstration of *EWSR1::PATZ1* fusion or *EWSR1* rearrangement is essential
Eosinophilic solid and cystic RCC	Solid and macrocystic growth; eosinophilic cells; cytoplasmic stippling	KRT20 frequently focal/patchy; cathepsin K commonly positive; KRT7 usually negative	Predominantly biallelic *TSC2* alteration	Usually indolent, although metastatic cases occur
Eosinophilic vacuolated tumor	Solid/nested oncocytic growth; prominent nucleoli; conspicuous intracytoplasmic vacuoles	CD117, cathepsin K, CD10, and GPNMB positive; KRT7 negative or focal; vimentin, HMB45, and Melan-A negative	*TSC1*, *TSC2*, or *MTOR* alteration	Usually indolent, but rare metastatic cases have been reported
TSC/MTOR-altered fibromyomatous RCC with hemangioblastoma-like areas	Fibromyomatous stroma with branching tubules/papillae; focal arborizing capillary network and stromal-cell–like areas	Conventional areas: KRT7 and CA9 positive; hemangioblastoma-like areas: inhibin-α and S100 positive and KRT7 negative; GPNMB positive in both	*TSC1*, *TSC2*, or *MTOR* alteration	Hemangioblastoma-like morphology represents part of the fibromyomatous RCC spectrum
Folliculin-mutated renal tumor	Biphasic or mosaic clear and eosinophilic cell populations; oncocytic/chromophobe-like morphology	PAX8 and GPNMB diffuse; KRT7, GATA3, and L1CAM often mosaic or variable	Biallelic *FLCN* inactivation	May indicate Birt–Hogg–Dubé syndrome; germline evaluation may be required
Renal oncocytic neoplasm, NOS/NCF	Low-grade oncocytic/eosinophilic morphology not fulfilling criteria for oncocytoma, chromophobe RCC, LOT, EVT, ESC RCC, sporadic FLCN-mutated neoplasm, or syndrome-associated oncocytic tumor	CD117 frequently positive but may be negative; KRT7 variable	No defining recurrent molecular alteration established	Diagnosis of exclusion for low-grade/low-risk oncocytic neoplasms; aggressive morphologic features and hereditary syndrome-associated tumors should exclude this designation

**Abbreviations**: 2SC, S-(2-succino)-cysteine; AMACR, α-methylacyl-CoA racemase; CA9, carbonic anhydrase 9; EMA, epithelial membrane antigen; FH, fumarate hydratase; FISH, fluorescence in situ hybridization; GPNMB, glycoprotein nonmetastatic melanoma protein B; NGS, next-generation sequencing; RCC, renal cell carcinoma; SDH, succinate dehydrogenase.

**Table 2:** Diagnostic, prognostic, and predictive roles of selected molecular biomarkers in renal tumors and the corresponding level of evidence.

Biomarker or Alteration	Principal Tumor Context	Biomarker Role	Analytical Method	Evidence Level	Current Clinical Implementation
VHL inactivation/3p loss	Clear cell RCC	Diagnostic and biologic; indirectly treatment-relevant	DNA sequencing, copy-number analysis, FISH	Established diagnostic biology; therapeutic relevance supported by prospective clinical evidence for downstream VEGF/HIF2α targeting	Molecular testing is not routinely required for diagnosis; pathway-directed therapies are established
BAP1 loss/alteration	Clear cell RCC	Prognostic; investigational predictive role	BAP1 IHC, DNA sequencing	Retrospective prognostic evidence; PARP inhibitor, radiation, and mTOR-response associations are predominantly preclinical or translational	Not a clinically validated treatment-selection biomarker
MET alteration	Papillary RCC	Diagnostic/supportive and potentially predictive	DNA sequencing, copy-number analysis	Prospective and retrospective clinical evidence varies according to alteration and therapeutic agent	May support enrollment in biomarker-selected trials or targeted treatment in selected settings
TFE3/TFEB alteration	TFE3-rearranged RCC and TFEB-altered RCC	Diagnostic; prognostic relevance may vary by alteration or fusion partner	Break-apart FISH, RNA-based NGS	Established diagnostic evidence; therapeutic implications remain investigational or based on limited clinical series	Used primarily for tumor classification
FH inactivation	FH-deficient RCC	Diagnostic, hereditary-risk, prognostic, and treatment-relevant	FH/2SC IHC, DNA sequencing, germline testing	Established diagnostic and hereditary evidence; germline and purely somatic *FH* inactivation both occur; prospective and retrospective therapeutic evidence is available for selected regimens	Routinely relevant to diagnosis; genetic counseling and germline evaluation are recommended because tumor findings alone cannot reliably distinguish hereditary from somatic-only disease
SDHx inactivation	SDH-deficient RCC	Diagnostic, hereditary-risk, and prognostic	SDHB IHC, DNA sequencing, germline testing	Established diagnostic and hereditary evidence; systemic treatment evidence is limited	Routinely relevant to diagnosis and genetic counseling
ALK rearrangement	ALK-rearranged RCC	Diagnostic and potentially predictive	ALK IHC, FISH, RNA-based NGS	Molecular diagnostic evidence is established; ALK IHC is a screening tool requiring molecular confirmation; treatment evidence is based predominantly on individual cases and small series	ALK-directed therapy may be considered in advanced disease, but predictive validation remains limited
TSC1/TSC2/MTOR alteration	ESC RCC, EVT, LOT, fibromyomatous RCC, and subsets of other RCCs	Diagnostic/supportive and potentially predictive	DNA-based NGS	Established biologic and diagnostic associations; predictive evidence for mTOR inhibitors is limited and heterogeneous	Not a universally validated treatment-selection biomarker
FLCN biallelic inactivation	Folliculin-mutated renal tumors/Birt–Hogg–Dubé syndrome	Diagnostic and hereditary-risk	DNA sequencing with copy-number analysis; germline testing	Emerging diagnostic evidence; established hereditary implications when germline	Should prompt consideration of genetic counseling and germline evaluation
SMARCB1 inactivation	SMARCB1-deficient renal medullary carcinoma	Diagnostic and prognostic	INI1 IHC, DNA/RNA-based testing	Established diagnostic association; therapeutic evidence remains limited	Routinely relevant to classification
HIF1A, EPAS1 (HIF2A), VEGFA/HIF–VEGF axis	Clear cell RCC	Biologic and prognostic; HIF2α is an established therapeutic target	HIF/VEGF immunohistochemistry; molecular profiling in research settings	Established clinical evidence for HIF2α-targeted therapy in selected RCC settings; limited evidence for the prognostic value of HIF/VEGF expression	HIF2α-targeted therapy is established in selected clinical settings; HIF/VEGF immunohistochemistry is not validated as a stand-alone predictive test for treatment selection

Evidence categories are descriptive rather than formal guideline grades. “Established” indicates reproducible diagnostic utility or support from prospective clinical data; “limited” indicates retrospective series or small clinical cohorts; and “investigational” indicates predominantly case-report-level, translational, or preclinical evidence. Identification of a therapeutically relevant alteration does not necessarily establish that alteration as a clinically validated predictive biomarker.

## Data Availability

No new data were created or analyzed in this study. Data sharing is not applicable to this article.
